# Case report: Early-onset Parkinson’s disease with lower limb spasticity in a new DJ-1/PARK7 patient

**DOI:** 10.3389/fnins.2024.1400001

**Published:** 2024-05-15

**Authors:** Masako Fujita, Haruo Nishijima, Atsuko Katagai, Chieko Suzuki, Nobutaka Hattori, Masahiko Tomiyama

**Affiliations:** ^1^Department of Neurology, Hirosaki University Graduate School of Medicine, Hirosaki, Japan; ^2^Department of Neurology, Faculty of Medicine, Juntendo University, Tokyo, Japan

**Keywords:** *DJ-1* variant, limb spasticity, Parkinson’s disease, parkinsonism, cognitive dysfunction

## Abstract

Rare autosomal recessive variants in *DJ-1*, a causative gene for early-onset Parkinson’s disease, have been associated with a variety of clinical syndromes in a limited number of patients. Here, we report a case of a novel *DJ-1* variant in a 39-year-old man with a 4-year history of parkinsonism, cognitive dysfunction, and lower limb spasticity. He was diagnosed with Parkinson’s disease. Genetic testing of the patient revealed compound heterozygous variants in the *DJ-1* gene (exon 6 deletion + c.242dup), of which exon 6 deletion was a novel variant. We conclude that variants in *DJ-1* should be considered possible causes of early-onset parkinsonism with spasticity and cognitive impairment, as in this case.

## 1 Introduction

Autosomal recessive Parkinson’s disease (ARPD) is associated with variants in *PRKN* (*PARK2*), *PINK1* (*PARK6*), and *DJ-1* (*PARK7*), which cause early-onset parkinsonism. The neuropathology of ARPD is characterized by selective degeneration of the substantia nigra and nucleus accumbens, with or without Lewy body pathology (Guadagnolo et al., 2021). Variants in *DJ-1* are rarely associated with ARPD; hence, the clinical characteristics of patients with such variants have not been conclusively established (Kasten et al., 2018). Pathological findings associated with *DJ-1* variants have only been reported in one autopsy report (Taipa et al., 2016). Herein, we report the case of a patient with levodopa-responsive parkinsonism, cognitive dysfunction, and lower extremity spasticity, harboring compound heterozygous variants in *DJ-1*, one of which is novel.

## 2 Case description

A 39-year-old Japanese man was referred to our hospital with early-onset parkinsonism. He was born to non-consanguineous parents without a family history of neurological diseases and had a healthy brother and sister. The patient’s medical history was unremarkable. At the age of 34, he developed a resting tremor in his right hand and manifested bradykinesia, postural instability, and recurrent falls. Two years later, he was diagnosed with PD at a local hospital and received levodopa treatment. At the age of 38, he developed cognitive impairment and levodopa-induced dyskinesia, after which he quit his job at a kindergarten.

## 3 Diagnostic assessment

Neurological examination revealed hypomimia, hypophonia, bradykinesia on finger and foot tapping, postural instability, and rigidity in all limbs and the trunk. The patient had a brisk tendon reflex in his lower limbs and a positive right Babinski sign. On examination, spastic gait was apparent in addition to a bilaterally reduced arm swing and a stooped bent posture (Supplementary Video 1). He had a Mini-Mental State Examination score of 14/30 and a Frontal Assessment Battery score of 6/18, indicating moderate cognitive decline. No abnormalities were observed in the cerebellar or sensory systems. Dystonia, anosmia, constipation, or rapid eye movement sleep behavior disorder was not noted. The patient’s routine blood and spinal fluid test results were normal. Brain magnetic resonance imaging revealed no abnormalities. Single-photon emission computed tomography revealed severely reduced uptake by the dopamine transporter in the basal ganglia. ^123^I-Metaiodobenzylguanidine (MIBG) myocardial scintigraphy showed normal uptake. In the levodopa challenge test, the patient’s Movement Disorder Society Unified Parkinson’s Disease Rating Scale Part III score improved from 58 to 47 points. Following levodopa administration, his short-stepped gait with frequent freezing improved, whereas his spastic gait became more noticeable. Skin biopsy showed no alpha-synuclein pathology. Sequencing of a panel of PD-related genes on a high-throughput next-generation sequencer using the Ione Torrent System (Thermo Fisher Scientific, Waltham, MA, United States) revealed single-nucleotide variants, followed by validation by Sanger sequencing and determination of allele frequencies using public databases. Copy number variants were analyzed by Multiplex Ligation-Dependent Probe Amplification (MLPA) using the SALSA MLPA® P051 Parkinson probe mix (MRC Holland, Amsterdam, the Netherlands). Compound heterozygous variants were found in the *DJ-1* gene (exon 6 deletion + c.242dup). Panel sequencing revealed no other putative pathogenic variants. Examination of the parents revealed that the father harbored a c.242dup and the mother harbored a deletion in exon 6 in the *DJ-1* gene. In the dbSNP database, the minor allele frequency of c.242dup was 0.00007%; exon 6 deletion has not been reported previously. The c.242dup variant was predicted to be pathological by *in silico* analysis of amino acids and was estimated to cause nonsense-mediated mRNA decay owing to the frameshift.

## 4 Discussion

Herein, we report a PD case with compound heterozygous variants, of which one is novel, in the *DJ-1* gene. The patient had levodopa-responsive parkinsonism, upper motor neuron signs, and cognitive dysfunction. In previous PD cases with *DJ-1* variants, upper motor neuron signs have been found in 27.5% of patients and cognitive dysfunction in 17.5% of patients (Supplementary Table 1). Although clinical signs are variable, levodopa reactivity is important for diagnosing *DJ-1* (*PARK7*) variant-associated ARPD.

Studies investigating the pathological variants in *DJ-1* are limited. Only a single autopsy case with a *DJ-1* variant showing Lewy bodies in the central nervous system has been reported (Taipa et al., 2016). In another case report of a *DJ-1* variant, a skin biopsy revealed α-synuclein pathology in tyrosine hydroxylase-positive fibers (Narendra et al., 2019). These findings suggest that *DJ-1* variants are involved in the development of synucleinopathies. Nevertheless, the results of the skin biopsy in our case did not suggest a peripheral synucleinopathy. Another diagnostic biomarker of Lewy body pathology is reduced MIBG uptake on MIBG myocardial scintigraphy. In our case, MIBG myocardial scintigraphy revealed no abnormalities. To the best of our knowledge, to date, only two studies have described MIBG myocardial scintigraphy in cases with *DJ-1* variants: one case exhibited decreased MIBG uptake, whereas the other case showed normal MIBG uptake (Quattrone et al., 2008). It is unclear whether all patients with *DJ-1* variants have Lewy body pathology. In an autopsy case with a *DJ-1* variant, neuronal loss in the substantia nigra and Lewy pathology resembled Braak stage 6. However, the dorsal vagus nucleus was relatively unaffected, whereas it is usually severely affected in sporadic cases of PD (Taipa et al., 2016). Lewy pathology progression in cases with a *DJ-1* variant may differ from that in sporadic cases. Our case suggested brain-first PD; the lack of autonomic disorder suggested weak peripheral involvement, whereas cognitive decline indicated cerebral extension. Negative skin biopsy and MIBG myocardial scintigraphy findings indicated that the peripheral systems were not yet affected; however, this could change with disease progression. Therefore, careful repeated follow-up examinations are warranted to monitor and clarify any potential neuropathological extension in the patient.

## Data availability statement

The datasets presented in this study can be found in online repositories. The names of the repository/repositories and accession number(s) can be found in the article/Supplementary material.

## Ethics statement

Written informed consent was obtained from the individual(s), and minor(s)’ legal guardian/next of kin, for the publication of any potentially identifiable images or data included in this article.

## Author contributions

MF: Conceptualization, Data curation, Formal analysis, Investigation, Methodology, Project administration, Validation, Writing – original draft, Writing – review & editing. HN: Conceptualization, Data curation, Formal analysis, Investigation, Methodology, Project administration, Supervision, Validation, Writing – review & editing, Writing – original draft. AK: Conceptualization, Investigation, Writing – review & editing. CS: Conceptualization, Investigation, Writing – review & editing. NH: Supervision, Writing – review & editing. MT: Conceptualization, Investigation, Supervision, Writing – review & editing.

## References

[ref1] Guadagnolo D. Piane M. Torrisi M. R. Pizzuti A. Petrucci S. (2021). Genotype-phenotype correlations in monogenic Parkinson disease: a review on clinical and molecular findings. Front. Neurol. 12:648588. doi: 10.3389/fneur.2021.648588, PMID: 34630269 PMC8494251

[ref2] Kasten M. Hartmann C. Hampf J. Schaake S. Westenberger A. Vollstedt E. J.. (2018). Genotype-phenotype relations for the Parkinson’s disease genes *Parkin*, *PINK1*, DJ1: MDSGene systematic review. Mov. Disord. 33, 730–741. doi: 10.1002/mds.27352, PMID: 29644727

[ref3] Narendra D. P. Isonaka R. Nguyen D. Schindler A. B. Kokkinis A. D. Ehrlich D.. (2019). Peripheral synucleinopathy in a *DJ1* patient with Parkinson disease, cataracts, and hearing loss. Neurology 92, 1113–1115. doi: 10.1212/WNL.0000000000007614, PMID: 31028127 PMC6556091

[ref4] Quattrone A. Bagnato A. Annesi G. Novellino F. Morgante L. Savettieri G.. (2008). Myocardial ^123^metaiodobenzylguanidine uptake in genetic Parkinson’s disease. Mov. Disord. 23, 21–27. doi: 10.1002/mds.21701 17975812

[ref5] Taipa R. Pereira C. Reis I. Alonso I. Bastos-Lima A. Melo-Pires M.. (2016). DJ-1 linked parkinsonism (PARK7) is associated with Lewy body pathology. Brain 139, 1680–1687. doi: 10.1093/brain/aww080, PMID: 27085187

