# Supplementary material for: Case report: Early-onset Parkinson’s disease with lower limb spasticity in a new DJ-1/PARK7 patient
Source: Front Neurosci. 2024 May 15;18:1400001. doi: 10.3389/fnins.2024.1400001 (PMC11138152; doi:10.3389/fnins.2024.1400001)
Supplement: Supplementary file 1 [file Table_1.DOCX]

Supplementary Material

# Supplementary Table

**Supplementary Table 1.** Summary of 41 published DJ-1 cases, with or without cognitive dysfunction and upper motor neuron signs

|  | **N** | **Mutation** | **Age of onset (years)** | **Cognitive dysfunction** | **Upper motor neuron sign** |
| --- | --- | --- | --- | --- | --- |
| **This Report** | **1** | **Deletion exon 6 + c.240-241insA: comp. het.** | **34** | **MMSE 14/30** | **Brisk tendon reflex in lower limbs, extensor plantar reflex** |
| **(Lesage et al., 2020)** | **1** | **p. Glu94*: homo** | **29** | **Normal** | **NA** |
| **(Lesage et al., 2020)** | **1** | **Thr154Ile + Thr154Ala: comp. het.** | **28** | **Normal** | **NA** |
| **(Narendra et al., 2019)** | **1** | **Deletion exon 4 + c.105dupT [p.Ala36Cysfs*12]: comp. het.** | **mid 20** | **NA** | **Extensor plantar reflex** |
| **(Stephenson et al., 2019)** | **1** | **c.90dupG: hom** | **28** | **Normal (MOCA 29/30)** | **NA** |
| **(Stephenson et al., 2019)** | **1** | **c.90dupG: hom** | **29** | **Normal (MOCA 28/30)** | **NA** |
| **(Taghavi et al., 2018)** | **1** | **p.Asp24Metfs*3: hom** | **27** | **NA** | **NA** |
| **(Di Nottia et al., 2017)** | **1** | **p.Thr154Lys: hom** | **mid 30s** | **Normal** | **NA** |
| **(Abbas et al., 2016)** | **1** | **p.Ile105Phe: hom** | **22** | **Normal** | **NA** |
| **(Erer et al., 2016)** | **1** | **c.322+4A>C: hom** | **23** | **NA** | **NA** |
| **(Taipa et al., 2016)** | **1** | **p.Leu172Gln: hom** | **22** | **Cognitive decline** | **Positive Babinski’s signs** |
| **(Hanagasi et al., 2016)** | **1** | **p.Gln45*: hom** | **24** | **MMSE 22/30** | **Upper (and lower) motor neuron sign** |
| **(Hanagasi et al., 2016)** | **1** | **p.Gln45*: hom** | **35** | **Normal (MMSE 28/30)** | **Upper (and lower) motor neuron sign** |
| **(Hanagasi et al., 2016)** | **1** | **p.Gln45*: hom** | **22** | **Normal (MMSE 29/30)** | **Upper (and lower) motor neuron sign** |
| **(Bras et al., 2014)** | **1** | **c.317_322+5delGTGCAGGTGAC: hom** | **15** | **Normal** | **Brisk tendon reflexes, extensor plantar reflex** |
| **(Darvish et al., 2013)** | **3** | **Deletion exon 5: hom** | **mean 36** | **NA** | **NA** |
| **(Ghazavi et al., 2011)** | **1** | **c.91-2A>G: hom** | **17** | **Normal** | **NA** |
| **(Ghazavi et al., 2011)** | **1** | **p.Ala107Pro: hom** | **22** | **Normal** | **NA** |
| **(Delva et al., 2021)** | **1** | **p.Pro158del: hom** | **5** | **Normal** | **NA** |
| **(Macedo et al., 2009)** | **1** | **p.Pro158del: hom** | **33** | **Cognitive problems** | **NA** |
| **(Tarantino et al., 2009)** | **1** | **c.252+8dupA + c.-24+66C>G: comp. het.** | **38** | **Normal (MMSE 29/30)** | **NA** |
| **(Guo et al., 2008)** | **1** | **p.Leu10Pro: hom** | **19** | **NA** | **No hyperreﬂexia** |
| **(Guo et al., 2008)** | **1** | **p.Leu10Pro: hom** | **18** | **NA** | **No hyperreﬂexia** |
| **(Annesi et al., 2005)** | **1** | **p.Glu163Lys: hom** | **36** | **Cognitive impairment** | **Upper (and lower) motor neuron sign** |
| **(Annesi et al., 2005)** | **1** | **p.Glu163Lys: hom** | **35** | **Cognitive impairment** | **Upper (and lower) motor neuron sign** |
| **(Annesi et al., 2005)** | **1** | **p.Glu163Lys: hom** | **24** | **Cognitive impairment** | **Upper (and lower) motor neuron sign** |
| **(Rajapakshe et al., 2023)** | **1** | **p.Glu163Lys: hom** | **17** | **Normal** | **Upper (and lower) motor neuron sign** |
| **(Hering et al., 2004)** | **1** | **p.Glu64Asp: hom** | **34** | **NA** | **NA** |
| **(Abou-Sleiman et al., 2003)** | **1** | **p.Met26Ile: hom** | **39** | **NA** | **NA** |
| **(Abou-Sleiman et al., 2003)** | **1** | **p.Met26Ile: hom** | **36** | **NA** | **NA** |
| **(Hague et al., 2003)** | **1** | **c.410-1G>C + p.Thr19Lysfs*5: comp. het.** | **24** | **NA** | **NA** |
| **(Dekker et al., 2003)** | **1** | **c.-4069_322+1852del: hom** | **40** | **NA** | **NA** |
| **(Dekker et al., 2003)** | **1** | **c.-4069_322+1852del: hom** | **31** | **NA** | **NA** |
| **(Dekker et al., 2003)** | **1** | **c.-4069_322+1852del: hom** | **NA** | **NA** | **NA** |
| **(Dekker et al., 2003)** | **1** | **c.-4069_322+1852del: hom** | **27** | **NA** | **NA** |
| **(Bonifati et al., 2003)** | **1** | **p.Leu166Pro: hom** | **28** | **NA** | **NA** |
| **(Bonifati et al., 2003)** | **1** | **p.Leu166Pro: hom** | **35** | **NA** | **NA** |
| **(Bonifati et al., 2003)** | **1** | **p.Leu166Pro: hom** | **27** | **NA** | **NA** |
| **Total** | **40** |  |  | **7/40 (17.5%)** | **11/40 (27.5%)** |
| **NA: Not available** | | | | | |

# Supplementary Video

**Supplementary Video 1.** The patient aged 39 years in the ON period exhibiting bradykinesia, rigidity, postural instability, hyperreflexia, spasticity in the lower limbs, positive right Babinski sign, and spastic gait.

# References

Abbas MM, Govindappa ST, Sudhaman S, Thelma BK, Juyal RC, Behari M, et al. Early onset Parkinson's disease due to DJ1 mutations: an Indian study. Parkinsonism Relat. Disord. (2016) 32:20-4. doi: 10.1016/j.parkreldis.2016.04.024

Abou-Sleiman PM, Healy DG, Quinn N, Lees AJ, Wood NW. The role of pathogenic DJ-1 mutations in Parkinson's disease. Ann Neurol*.* (2003) 54:283-6. doi: 10.1002/ana.10675

Annesi G, Savettieri G, Pugliese P, D'Amelio M, Tarantino P, Ragonese P, et al. DJ-1 mutations and parkinsonism-dementia-amyotrophic lateral sclerosis complex. Ann. Neurol. (2005) 58:803-7. doi: 10.1002/ana.20666

Bonifati V, Rizzu P, van Baren MJ, Schaap O, Breedveld GJ, Krieger E, et al. Mutations in the DJ-1 gene associated with autosomal recessive early-onset parkinsonism. Science. (2003) 299:256-9. doi: 10.1126/science.1077209

Bras JM, Guerreiro RJ, Teo JTH, Darwent L, Vaughan J, Molloy S, et al. Atypical parkinsonism-dystonia syndrome caused by a novel DJ1 mutation. Mov. Disord. Clin. Pract. (2014) 1:45-9. doi: 10.1002/mdc3.12008

Darvish H, Movafagh A, Omrani MD, Firouzabadi SG, Azargashb E, Jamshidi J, et al. Detection of copy number changes in genes associated with Parkinson's disease in Iranian patients. Neurosci. Lett. (2013) 551:75-8. doi: 10.1016/j.neulet.2013.07.013

Dekker M, Bonifati V, van Swieten J, Leenders N, Galjaard RJ, Snijders P, et al. Clinical features and neuroimaging of PARK7-linked parkinsonism. Mov. Disord. (2003) 18:751-7. doi: 10.1002/mds.10422

Delva A, Race V, Boon E, Van Laere K, Vandenberghe W.Parkinson's disease with a homozygous PARK7 mutation and clinical onset at the age of 5 years. Mov. Disord. Clin. Pract. (2021) 8:149-52. doi: 10.1002/mdc3.13114

Di Nottia M, Masciullo M, Verrigni D, Petrillo S, Modoni A, Rizzo V, et al. DJ-1 modulates mitochondrial response to oxidative stress: clues from a novel diagnosis of PARK7. Clin. Genet. (2017) 92:18-25. doi: 10.1111/cge.12841

Erer S, Egeli U, Zarifoglu M, Tezcan G, Cecener G, Tunca B,et al. Mutation analysis of the PARKIN, PINK1, DJ1, and SNCA genes in Turkish early-onset Parkinson's patients and genotype-phenotype correlations. Clin. Neurol. Neurosurg. (2016) 148:147-53. doi: 10.1016/j.clineuro.2016.07.005

Ghazavi F, Fazlali Z, Banihosseini SS, Hosseini SR, Kazemi MH, Shojaee S, et al. PRKN, DJ-1, and PINK1 screening identifies novel splice site mutation in PRKN and two novel DJ-1 mutations. Mov. Disord. (2011) 26:80-9. doi: 10.1002/mds.23417

Guo JF, Xiao B, Liao B, Zhang XW, Nie LL, Zhang YH, et al. Mutation analysis of Parkin, PINK1, DJ-1 and ATP13A2 genes in Chinese patients with autosomal recessive early-onset Parkinsonism. Mov. Disord. (2008) 23:2074-9. doi: 10.1002/mds.22156

Hague S, Rogaeva E, Hernandez D, Gulick C, Singleton A, Hanson M, et al. Early-onset Parkinson's disease caused by a compound heterozygous DJ-1 mutation. Ann. Neurol. (2003) 54:271-4. doi: 10.1002/ana.10663

Hanagasi HA, Giri A, Kartal E, Guven G, Bilgiç B, Hauser AK, et al. A novel homozygous DJ1 mutation causes parkinsonism and ALS in a Turkish family. Parkinsonism Relat. Disord. (2016) 29:117-20. doi: 10.1016/j.parkreldis.2016.03.001

Hering R, Strauss KM, Tao X, Bauer A, Woitalla D, Mietz EM, et al. Novel homozygous p.E64D mutation in DJ1 in early onset Parkinson disease (PARK7). Hum. Mutat*.* (2004) 24:321-9. doi: 10.1002/humu.20089

Lesage S, Lunati A, Houot M, Romdhan SB, Clot F, Tesson C, et al. Characterization of recessive Parkinson disease in a large multicenter study. Ann. Neurol. (2020) 88:843-50. doi: 10.1002/ana.25787

Macedo MG, Verbaan D, Fang Y, van Rooden SM, Visser M, Anar B, et al. Genotypic and phenotypic characteristics of Dutch patients with early onset Parkinson's disease. Mov. Disord. (2009) 24:196-203. doi: 10.1002/mds.22287

Narendra DP, Isonaka R, Nguyen D, Schindler AB, Kokkinis AD, Ehrlich D, et al. Peripheral synucleinopathy in a DJ1 patient with Parkinson disease, cataracts, and hearing loss. Neurology (2019) 92:1113-5. doi: 10.1212/wnl.0000000000007614

Rajapakshe I, Mulroy E, Magrinelli F, Makawita C, Bhatia KP, Senanayake B. Cranial dystonia as an isolated presentation of DJ-1 disease: case report and literature review. Mov. Disord. Clin. Pract. (2023) 10:313-5. doi: 10.1002/mdc3.13591

Stephenson SE, Djaldetti R, Rafehi H, Wilson GR, Gillies G, Bahlo M, et al. Familial early onset Parkinson's disease caused by a homozygous frameshift variant in PARK7: clinical features and literature update. Parkinsonism Relat Disord. (2019) 64:308-11. doi: 10.1016/j.parkreldis.2019.03.013

Taghavi S, Chaouni R, Tafakhori A, Azcona LJ, Firouzabadi SG, Omrani MD, et al. A clinical and molecular genetic study of 50 families with autosomal recessive parkinsonism revealed known and novel gene mutations. Mol. Neurobiol. (2018) 55:3477-89. doi: 10.1007/s12035-017-0535-1

Taipa R, Pereira C, Reis I, Alonso I, Bastos-Lima A, Melo-Pires M, et al. DJ-1 linked parkinsonism (PARK7) is associated with Lewy body pathology. Brain (2016) 139:1680-7. doi: 10.1093/brain/aww080

Tarantino P, Civitelli D, Annesi F, De Marco EV, Rocca FE, Pugliese P, et al. Compound heterozygosity in DJ-1 gene non-coding portion related to parkinsonism. Parkinsonism Relat. Disord. (2009) 15:324-6. doi: 10.1016/j.parkreldis.2008.07.001
